# Identifying community thresholds for lotic benthic diatoms in response to human disturbance

**DOI:** 10.1038/s41598-017-04445-7

**Published:** 2017-06-23

**Authors:** Tao Tang, Ting Tang, Lu Tan, Yuan Gu, Wanxiang Jiang, Qinghua Cai

**Affiliations:** 1 State Key Laboratory of Freshwater Ecology and Biotechnology, Institute of Hydrobiology, Chinese Academy of Sciences, Wuhan, 430072 China; 2 College of Life Sciences, Zaozhuang University, Zaozhuang, 277160 China

## Abstract

Although human disturbance indirectly influences lotic assemblages through modifying physical and chemical conditions, identifying thresholds of human disturbance would provide direct evidence for preventing anthropogenic degradation of biological conditions. In the present study, we used data obtained from tributaries of the Three Gorges Reservoir in China to detect effects of human disturbance on streams and to identify disturbance thresholds for benthic diatoms. Diatom species composition was significantly affected by three in-stream stressors including TP, TN and pH. Diatoms were also influenced by watershed % farmland and natural environmental variables. Considering three in-stream stressors, TP was positively influenced by % farmland and % impervious surface area (ISA). In contrast, TN and pH were principally affected by natural environmental variables. Among measured natural environmental variables, average annual air temperature, average annual precipitation, and topsoil % CaCO_3_, % gravel, and total exchangeable bases had significant effects on study streams. When effects of natural variables were accounted for, substantial compositional changes in diatoms occurred when farmland or ISA land use exceeded 25% or 0.3%, respectively. Our study demonstrated the rationale for identifying thresholds of human disturbance for lotic assemblages and addressed the importance of accounting for effects of natural factors for accurate disturbance thresholds.

## Introduction

Threshold responses of organisms along environmental gradients are commonly observed in streams. That is, relatively small changes in environmental conditions can induce substantial or sometimes catastrophic changes in structure and function of lotic communities^1, 2^. Such thresholds can provide important information on sensitivity/tolerance amplitudes and breakpoints of organisms to environmental alterations, which are very helpful for setting specific management targets for streams^3–5^. Therefore, identifying thresholds has become an emerging field in stream ecology^6^.

Benthic algae are the principal primary producers in streams with high sensitivity to in-stream stressors including excess nutrient loadings and habitat degradation^7^, and are extensively used for threshold identification. Among algae-based thresholds, using nutrient-algal relationship to infer nutrient thresholds (such as total phosphorus [TP] and total nitrogen [TN]) has been highly addressed because nutrients have significant effects on algal species composition, and excess nutrients may lead to algal blooms when other environmental conditions are favorable^8–10^. Nutrient thresholds for algae have been increasingly applied in nutrient criteria development^11–14^.

However, only detecting algal responses to nutrients may be not enough and may lead to over- or under-protection problems because in-stream nutrient concentrations are influenced by both natural and anthropogenic factors^15, 16^. Conceptual models suggested that regional natural factors, including climate, geology, and soils are important ultimate factors acting as landscape filters to constrain local-scale physical and chemical factors such as water temperature, nutrients, sediments, and shear stress^17^. Algal compositions in streams are more directly affected by local factors than regional factors^18^. Besides natural factors, Human activities also have significantly influence on lotic algal assemblages by modifying physical habitat, water chemistry, and hydrological characteristics of streams^19–21^. Although nutrient thresholds can provide important information on algal responses to nutrient changes, such thresholds may not be effective in associating degradation of algal conditions with human disturbance, which is essential for management practices. It is likely that a nutrient threshold is mainly determined by effects of natural factors on algal assemblages when natural environments are highly heterogeneous.

In contrast, although human activities do not directly affect algal assemblages, they are the original source of numerous stressors and can be viewed as a composite stressor^22^. So, detecting thresholds of human disturbance based on disturbance-algal response relationships would be especially useful in providing direct evidence for managing anthropogenic degradation of streams. Few studies have documented human disturbance-algal response relationships. For instance, Zampella *et al*. found significant differences in diatom species compositions between developed/agricultural stream sites and cranberry and forest stream sites in USA^23^. Lavoie *et al*. found that values of the Eastern Canadian Diatom Index (IDEC) were systematically low in watersheds with high proportion of farmland. Additionally, urban centers and industrial activities also had significant contributions to pollution which resulting in low IDEC values^24^. Threshold responses were also detected for lotic algal metrics to the percentage of agriculture, pasture, and impervious cover in the watershed^25, 26^. However, potential influence of natural factors on algal metrics has not been fully considered when identifying thresholds of human disturbance. This is especially true for estimating community thresholds because the commonly used method was designed to explore changes in the species composition and structure along one environmental gradient (e.g., Threshold Indicator Taxa Analysis [TITAN])^27^. Community thresholds are derived from taxon-specific responses along environmental gradients thus may provide more comprehensive information than univariate metrics (e.g., biodiversity indices, sensitive/tolerance indicators). Therefore, it is valuable to explore methods that can identify the community threshold to human disturbance while accounting for confounding effects of natural factors.

In the present study, we use data obtained from tributaries of the Three Gorges Reservoir (TGR) in China to identify human land use thresholds for benthic diatom assemblages. We have two objectives: (1) specifying significant relationships among human land use, natural environmental variables, in-stream stressors, and diatom species composition. We hypothesize that algal communities are directly influenced by in-stream stressors including nutrients; however, some of these stressors might principally related to natural backgrounds other than human land use. Testing this hypothesis provides rationale for identifying land use thresholds. And, (2) identifying community thresholds for benthic diatoms in response to human land use while accounting for effects of natural factors. We postulate that substantial compositional changes would occur in diatom assemblages when human land use exceeds a certain proportion in the watershed. Meanwhile, diatom assemblages also display threshold responses to natural factors, which would blur disturbance-algal response relationships if not considered.

## Methods

### Study region, sample collecting and processing

Our study was conducted in tributaries of the TGR. This region is located in the western-central part of China, belonging to the subtropical humid monsoon climate zone with distinct seasonality of wet and hot summers and dry and cold winters. Average annual precipitation in the TGR region is 1100–1500 mm with an average annual air temperature of 12–19 °C^28^. Purple sand shale and limestone are the principal bedrocks of this mountainous region, with common soil types of purple soil, calcareous soil, yellow soil and paddy soil^29^.

**Figure 1:**
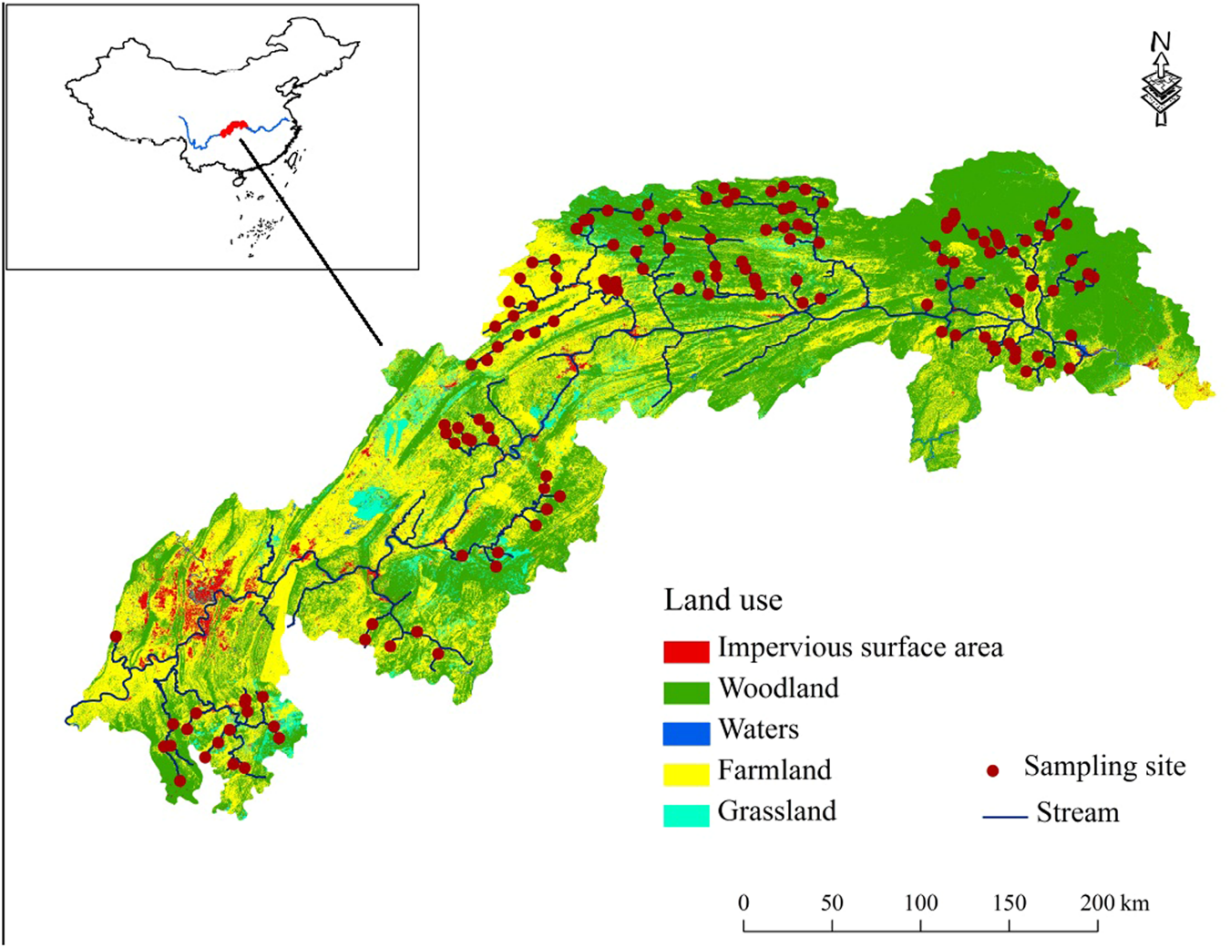
Location of the sampling sites and land use patterns in the Three Gorges Reservoir region. The map was generated using ArcGIS 10.0 (ESRI, Redlands, CA, USA: http://www.esri.com/software/arcgis).

Benthic diatoms, water chemistry, and landscape attributes were surveyed from 149 sites located in 23 tributaries of the TGR in April to May of 2015 (Fig. 1). Sites located in both headwaters and downstream were selected with regard to accessibility and probability to represent broad ranges of both human activities and natural environmental backgrounds (elevation ranging from 188–2295 m among sites). The interval between any two sites was larger than 10 km to avoid spatial dependence. At each site, 12 stones with diameter <20 cm were haphazardly picked up from the riverbed along a 100-m long reach, and benthic diatoms were sampled from the stone surfaces using a circular lid (radius: 2.5 cm) as the area delimiter. For each stone, the surface within the lid was vigorously scrubbed using a nylon brush and fully rinsed with distilled water. All 12 subsamples were combined into one composite sample for each site and preserved in 4% formalin in the field. The samples were transported back to the laboratory, and diatoms were cleaned by acid for mounting on microscope slides using neutral balsam (Hushi brand, Sinopharm Chemical Reagent Co., Ltd, China). Diatoms were enumerated and identified using a compound microscope (Olympus CX21: Olympus Optical Co., Japan) at 1000X. At least 600 valves were identified to the species or variety level following the taxonomic references^30–36^, and relative abundance for each taxon within each site was calculated.

For each site, geographic coordinates and elevation were recorded using a hand-held GPS receiver (Magellan 315). Conductivity, pH, and turbidity were measured with a HACH portable water quality monitoring meter in the field. Water sample was collected and preserved in a 350-ml polyethylene bottle by adding concentrated sulfuric acid till pH < 2. In the laboratory, TP and TN were determined on a Skalar segmented flow analyzer (Skalar Analytical B.V., The Netherlands).

Land use data were derived from Landsat 8 satellite images with a resolution of 30 × 30 m taken in 2014 and 2015. Land use in the TGR region was classified into 5 categories: woodland, grassland, farmland, impervious surface area (ISA, including towns, roads, and other man made facilities), and waters. The percentage of each land use category in the upstream watershed was calculated for each study site. Four climate variables were derived from WorldClim (http://www.worldclim.org), namely average annual air temperature (temp_annual), average April air temperature (temp_4), average annual precipitation (prec_annual), and average April precipitation (prec_4) in the upstream watershed for each site. The mean of the soil’s physical and chemical properties in the upstream watershed for each site was determined from the 1:1 million scaled soil database of China^37^. Although there are plenty of data on soil properties of different layers in this database, we only used 11 topsoil (0–30 cm) properties that are not likely to be influenced by human activities to represent the natural backgrounds for each site. These properties were volume percentage gravel (% gravel), percentage sand (% sand), percentage silt (% silt), percentage clay (% clay), reference bulk density (ref_bulk), cation exchange capacity of the clay fraction (CEC_clay), cation exchange capacity (CEC_soil), base saturation (BS), total exchangeable bases (TEB), calcium carbonate (lime) content (% CaCO_3_), and electrical conductivity (ECE). We used soil properties in our study based on data availability. Tang *et al*. found that soil characteristics in this region were highly correlated with bedrock geology and were not significantly affected by human activities^38^. Therefore, these soils properties can be regarded as surrogates for bedrock geology.

### Data analysis

We first used structural equation modeling (SEM) to evaluate the effects of human and natural factors on in-stream stressors and diatom species composition. SEM is a statistical method that has been increasingly used to test hypotheses concerning causal relationships in ecological studies^39^. We used the % farmland and % ISA in the watershed to represent human disturbance, with the 4 climate variables and the 11 soil variables as natural factors. Five water chemistry variables, i.e., TN, TP, pH, conductivity, and turbidity, were selected as in-stream stressors. These in-stream stressors have important effects on spatio-temporal patterns of lotic benthic algal assemblages and their values are strongly related to watershed human activities^40, 41^. Prior to the SEM analysis, non-percentage variables were log(x + 1) transformed with arcsine square root transformation for percentage data to reduce the influence of extreme values on the analysis. Spearman correlations between variables within each variable category (i.e., land use, natural factors, and in-stream stressors) were thereafter analyzed, which indicated that temp_4, prec_4, % silt, CEC_clay, CEC_soil, BS, and ECE were highly correlated with other natural environmental variables (ρ > 0.75). These 7 natural environmental variables were therefore excluded from further analysis. Diatom species composition was indicated by relative abundance. We used reduced-multidimensional data rather than original relative abundance matrices in the analysis because SEM can only handle one-dimensional variables. Nonmetric multidimensional scaling (NMDS) ordination based on Bray-Curtis dissimilarity of relative abundance of diatom taxa between sites was performed to produce reduced-dimensional data. NMDS is a method of projecting objects from multidimensional distance matrices (e.g., dissimilarities of species composition between sampling sites) onto a space of minimized dimensionality while the rank order of these objects is conserved as much as possible^42^. In our study, NMDS was conducted with the R function “monoMDS” in the package “vegan”, and the first two NMDS axis scores (NMDS1 and NMDS2, with a stress metric of 0.19) for each site were used as reduced-multidimensional data for diatom species composition. Relative abundance data were arcsine square root transformed for the NMDS analysis to reduce the influence of dominant species.

We performed SEM with following steps^39^. (1) Proposing the conceptual model. We hypothesized that both human activities and natural factors indirectly affect benthic diatom species composition through specific pathways to in-stream stressors. (2) Specifying the model. The 2 land use and the 8 natural environmental variables were exogenous variables in the model; with the 5 in-stream stress variables and the 2 diatom NMDS axes scores as endogenous variables. The stress variables were influenced by all exogenous variables, while the diatom metrics were only influenced by the stress variables. (3) Identifying significant pathways for the model. To achieve a parsimonious model with satisfactory fits, all non-significant pathways in the initial model was dropped (with *P* > 0.05) successively. Additionally, modification indices, i.e. expected decrease in χ^2^ for including an additional path in the model, were used to detect significant (χ^2^ > 3.84) and ecologically meaningful pathways not considered previously during parameter estimation. (4) Assessing the model fit. Overall model fit was estimated with χ^2^ statistics, root mean square error of approximation (RMSEA), and the comparative fit index (CFI). Non-significant χ^2^ statistics with *P* > 0.05 indicate that the fitted model agrees with the hypothesis. RMSEA approaches 0 and CFI value close to 1.0 also indicate a good fit of the model. SEM was conducted with R package “lavaan”.

After identifying important human land use and natural environmental variables by using SEM, we performed gradient forest (GF) to calculate community-level thresholds of these variables for benthic diatoms. GF is a novel nonparametric method for exploring the magnitude and shape of compositional changes in the community along environmental gradients^43, 44^. This method is an extension version of random forest (RF), which is a regression tree based approach with bootstrapping procedure to estimate predictor importance for one response variable and detect the response curve along environmental gradients^45^. For a given community with a set of predictors, GF begins with RF modeling response of each taxon along each predictor gradient. RF results are aggregated to calculate the cumulative and overall importance of each predictor together with a cumulative importance value *R* ^*2*^ (i.e. the aggregated value of goodness-of-fit). Only taxa with *R* ^*2*^ > 0 in RF models are included in the final GF model. The community-level importance of predictors is assessed, and empirical shape and magnitude of compositional changes along individual predictor gradients are fitted. The response thresholds (change points) are visually estimated by using response curves, in which blue lines representing the ratios between binned raw importance density and densities of observed predictor values (red lines) and the horizontal dashed lines representing ratios = 1 were both plotted^43^. For each predictor, we identified the peak of the standardized split density as the threshold, with the cross points between the blue and dashed lines where subsequent ratios >1 as the threshold range^46^. For GF modeling, we used arcsine square root transformed relative abundance data of the diatom species composition as the response matrix and % farmland, % ISA of land use and important natural environmental variables as predictors. No predictor was transformed because GF is a highly flexible machine learning method that is insensitive to outliers. GF modeling was carried out with R package “gradientForest”.

## Results

### Description of watershed land use, natural factors, in-stream stressors, and diatoms

There were substantial differences in human land use, natural environmental variables, and in-stream stressors among the study sites (Table 1). Agriculture was the dominant human land use in the study sites where averagely 29.5% of land was covered by farmland with the range of 0 to 99.5% among the sites. By comparison, only 0–2.6% of land was occupied by ISA with the mean coverage of 0.4%. Mean annual precipitation and air temperature varied from 855 to 1446 mm and 5.6–18.9 °C, respectively. Soil properties also varied considerably among the sites, and the maximum and minimum values for most of the soil variables relatively differed by approximately 2–3 times. Nutrient concentrations ranged from 0.102 to 8.025 mg TN/L and 0.008 to 0.423 mg TP/L, with mean concentrations of 1.491 mg TN/L and 0.059 mg TP/L. Conductivity varied between 25.4 and 700 μS/cm, with the mean of 241.3 μS/cm. By contrast, turbidity was low in most sites, with the mean of 3.34 NTU. Water pH varied from neutral to slightly alkaline, with the mean of 8.3.

**Table 1:** Description and statistics of variables measured for the study sites located in tributaries of the Three Gorges Reservoir.

Variable	Description/Unit	Class	Min	Mean	Max
% farmland	Percent watershed farmland (%)	L	0	29.5	99.5
% ISA	Percent watershed impervious surface area (%)	L	0	0.4	2.6
prec_annual	Mean annual precipitation (mm)	N	855	1118	1446
prec_4	April mean precipitation (mm)	N	60	97	162
temp_annual	Mean annual air temperature (°C)	N	5.6	16.0	18.9
temp_4	April mean air temperature (°C)	N	5.6	16.2	19.3
% gravel	Percent gravel content in topsoil (%vol.)	N	4.0	8.7	19.0
% sand	Percent sand fraction in topsoil (%wt.)	N	31.0	38.8	45.1
% silt	Percent silt fraction in topsoil (%wt.)	N	22.0	36.6	44.0
% clay	Percent clay fraction in topsoil (%wt.)	N	19.9	24.6	47.0
ref_bulk	Soil reference bulk density in topsoil (kg/dm^3^)	N	1.26	1.38	1.42
CEC_clay	Cation exchange capacity of the clay fraction in topsoil (cmol/kg)	N	30.0	37.7	53.0
CEC_soil	Cation exchange capacity in topsoil (cmol/kg)	N	10.9	13.9	27.0
BS	Base saturation in topsoil (%)	N	35.3	66.2	100.0
TEB	Total exchangeable bases in topsoil (cmol/kg)	N	4.3	11.2	26.8
% CaCO_3_	Percent calcium carbonate content in topsoil (%wt.)	N	0.0	1.7	15.0
ECE	Mean electrical conductivity of topsoil (dS/m)	N	0.1	0.1	0.9
TN	Total nitrogen (mg/L)	S	0.102	1.491	8.025
TP	Total phosphorus (mg/L)	S	0.008	0.059	0.423
Cond	Conductivity (μS/cm)	S	25.4	241.3	700
pH		S	7.1	8.3	9.4
Turb	Turbidity (NTU)	S	0.18	3.34	41.90

Variables were categorized into three classes: land use variables (L), natural environmental variables (N), and in-stream stressors (S), with the minimum (Min), arithmetic mean (Mean), and maximum (Max) presented. (%vol.: volume percentage; %wt.: weight percentage).

One hundred and forty-six diatom taxa were observed in the 149 study sites, among which 8 taxa had mean relative abundance higher than 1%. *Achnanthidium minutissimum* (Kützing) Czarnecki was the most abundant taxon occurring in 147 sites with the mean relative abundance of 42.2%. *Cocconeis placentula* Ehrenberg was the second most dominant taxon occurring in 145 sites with the relative abundance of 26.2%. *Gomphoneis heterominuta* Mayama & Kawashima, *Navicula cryptotenella* Lange-Bertalot, *Gomphonema angustatum* (Kützing) Rabenhorst, *Encyonema caespitosum* var. *pediculus* (Ehrenberg) De Toni, and *Planothidium lanceolatum* (Brébisson ex Kützing) Lange-Bertalot were also observed in more than 50% of the sites. The number of taxa observed in each site varied between 5 and 30, with the mean number of 15.

### Significant relationships

**Figure 2:**
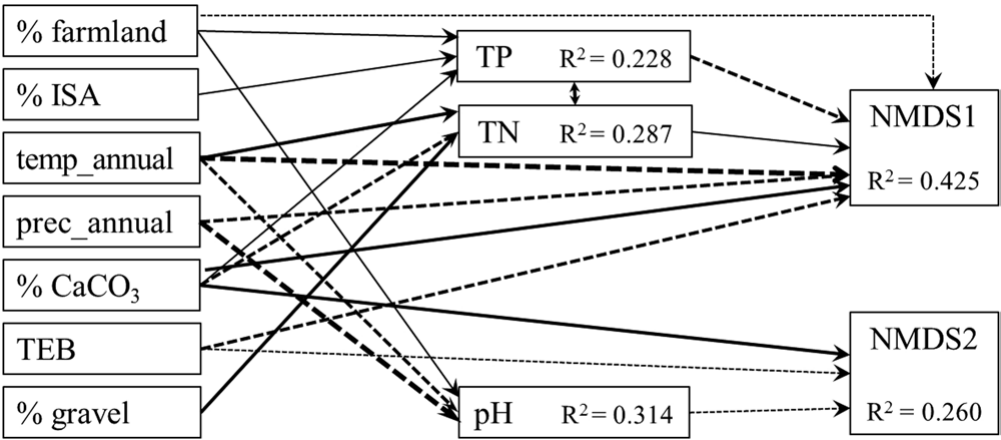
Structural equation modeling identifying significant pathways among human land use, natural environmental variables, in-stream stressors, and lotic benthic diatom species composition (χ^2^ = 0.350, RMSEA = 0.024, CFI = 0.993). Solid lines indicate positive standardized path coefficients (r), and dotted lines indicate negative ones. Thicker lines represent stronger relationships (—: |r| < 0.3; **─**: 0.3 ≤ |r| < 0.5; **▬**: |r| ≥ 0.5). For the full names of each variable, please see Table 1.

Significant relationships among human land use, natural environmental variables, in-stream stressors, and diatom species composition were detected with SEM (Fig. 2). Species composition of benthic diatoms was significantly affected by three in-stream stressors including TP, TN and pH. Watershed % farmland and natural environmental variables of temp_annual, prec_annual, % CaCO_3_, and TEB also had direct effects on diatoms. Regarding the three important in-stream stressors, TP was positively affected by watershed % farmland, % ISA, and % CaCO_3_ (Fig. 2). Conversely, TN concentration was solely determined by natural environmental variables, i.e., temp_annual, % CaCO_3_, and % gravel. pH was also principally influenced by temp_annual and prec_annual, with minor effects by % farmland. SEM detected a significant covariate relation between TP and TN (Fig. 2).

### Thresholds and indicator species for human disturbance

**Figure 3:**
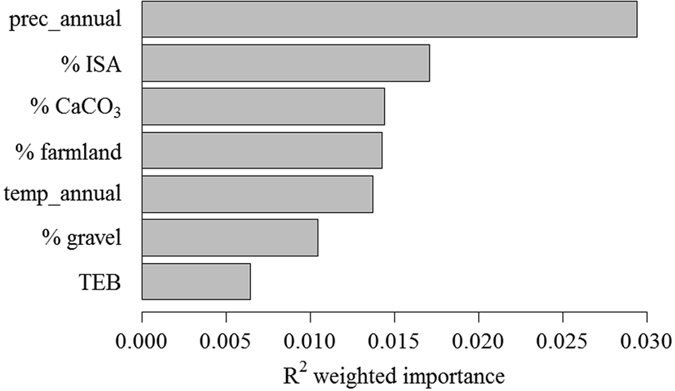
Overall R^2^ weighted importance of human land use and natural environmental variables for species composition of benthic diatom assemblages in tributaries of the Three Gorges Reservoir. For the full names of each variable, please see Table 1.

**Figure 4:**
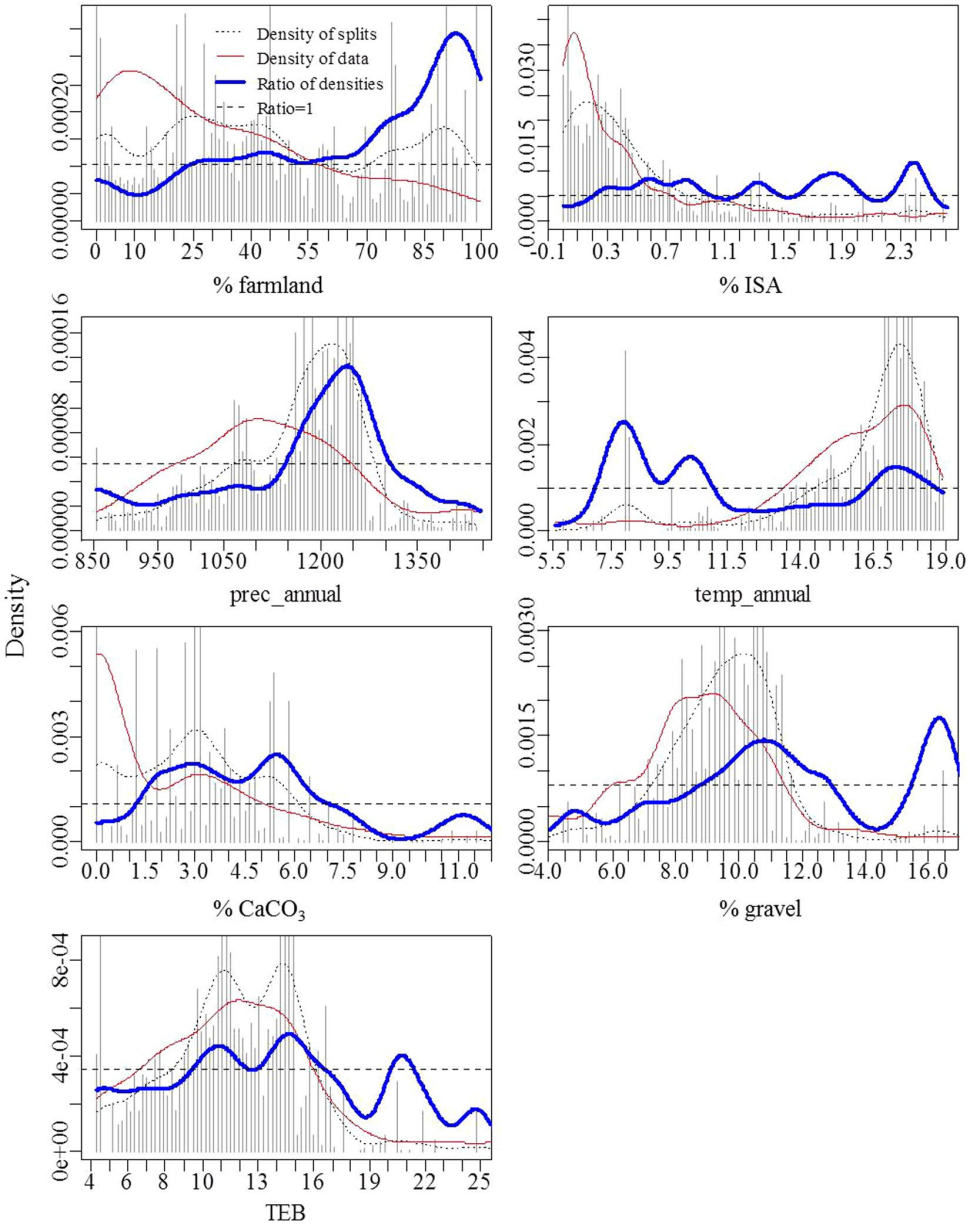
Compositional changes of benthic diatom assemblages across individual gradient of human land use and important natural environmental variables in tributaries of the Three Gorges Reservoir. Blue lines indicate ratios between binned raw importance density (gray vertical lines) and densities of observed predictor values (red lines) with horizontal dashed lines representing ratios = 1. Ratios >1 indicate steep changes. The cross points between the blue and dashed lines where subsequent ratios >1 were identified as thresholds. For the full names of each variable, please see Table 1.

Gradient forest modeled relationships among diatom species composition and the significant human and natural environmental variables detected by SEM. The overall conditional importance measures showed that prec_annual was the most important predictor (Fig. 3), followed by % ISA, % CaCO_3_, % farmland, and temp_annual. In contrast, % gravel and TEB were the least important predictors to diatom assemblages. GF also modeled response curves of diatom species composition along each predictor gradient (Fig. 4). As for human land use variables, diatoms displayed a “no tolerance” response to % ISA that a steep compositional change occurred at approximately 0.3% ISA. The diatom species composition continuously changed when % ISA was higher than this threshold and had the steepest change at approximately 2.4% ISA. On the contrary, diatom assemblages displayed a relatively higher tolerance to agricultural disturbance that a substantial change in species composition occurred at approximately 25% farmland in the watershed. Percentages of farmland beyond this threshold always had important impacts on diatoms, with 90% farmland leading to the steepest change in diatom compositions. Compositional changes in diatom assemblages also observed along gradients of the 5 natural environmental variables. Specifically, threshold responses for diatom assemblages occurred at about 1250 m of mean annual precipitation (with the threshold range of 1150–1300 m), 7.5 °C of mean air temperature (7.0–11.0 °C) and another minor peak of 17.0 °C (16.0–19.0 °C), 6.0% of CaCO_3_ content (1.2–7.0%), 11.0 and 16.0% of gravel content (9.0–13.0% and >15.5%), and 15 cmol/kg TEB (9.5–17 cmol/kg), respectively.

**Figure 5:**
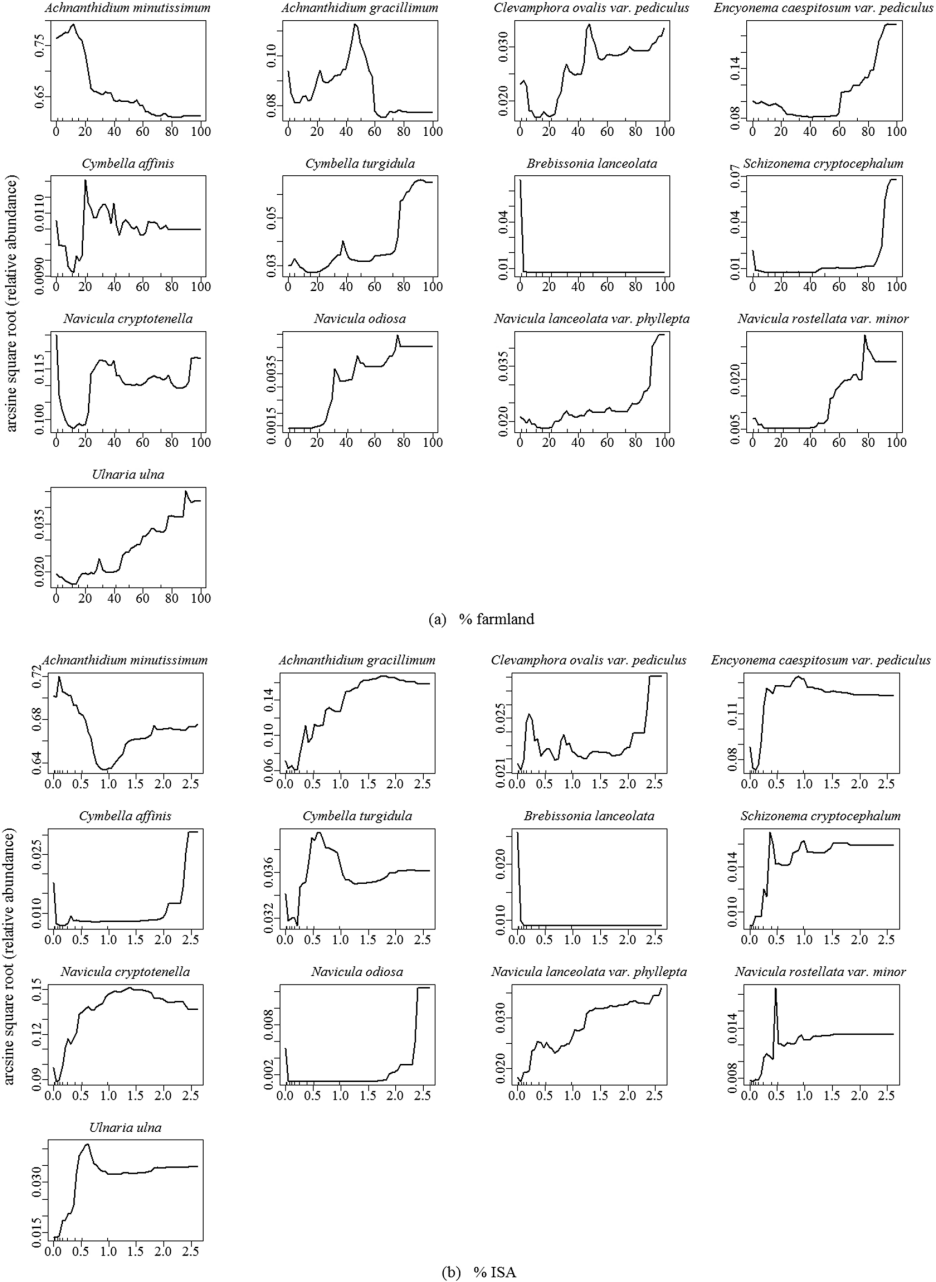
Partial dependence plots for random forest predictions of changes in relative abundance of selected diatom taxa across the gradient of watershed % farmland (**a**) and % impervious surface area (% ISA) (**b**) in tributaries of the Three Gorges Reservoir.

There were 13 taxa had high cumulative importance values (R^2^ > 0.10) across all predictor gradients. Taxa-specific changes in relative abundance across human land use gradients were observed by checking partial dependence plots of random forest predictions in which effects of natural environmental variables were controlled as their means (Fig. 5a,b). Relative abundance of *Brebissonia lanceolata* (Agardh) Mahoney and Reimer sharply decreased once % farmland or ISA % began to increase. *A. minutissimum* was also sensitive to human land use and its relative abundance decreased considerably when farmland reached 20%. This species displayed a U-shape response curve to % ISA that its relative abundance decreased at low % ISA but increased when % ISA was high. Eight species became abundant when % farmland or % ISA increased, i.e.: *Clevamphora ovalis* var*. pediculus* (Kützing) Mereschkowsky, *Encyonema caespitosum* var. *pediculus* (Ehrenberg) De Toni, *Cymbella turgidula* Grunow, *Schizonema cryptocephalum* (Kützing) Kuntze, *Navicula odiosa* Wallace, *Navicula lanceolata* var. *phyllepta* (Kützing) Van Heurck, *Navicula rostellata var. minor* Tempère & Peragallo, and *Ulnaria ulna* (Nitzsch) Compère. Two species, *Achnanthidium gracillimum* (Meister) Mayama in Kobayasi and *Cymbella affinis* Kützing, were more abundant in sites with 20–50% and 20–40% farmland land use, respectively. In contrast, their relative abundance increased along the ISA gradient. *Navicula cryptotenella* Lange-Bertalot showed U-shape responses to the farmland gradient; while, it was more abundant with increasing ISA.

## Discussion

We found that TP, TN, and pH were important in-stream stressors that directly affected diatom species composition in tributaries of the TGR. This result is similar to the findings of others^40, 47^. Nevertheless, distinct relationships were detected between these variables and human and natural factors in our study. TP was mainly determined by both % farmland and % ISA in the watershed. Conversely, TN and pH were principally attributed to natural backgrounds. We checked frequency distributions of TN concentrations and found that the median TN concentration was 1.15 mg/L and 75% of sites had TN concentrations less than 2.0 mg/L. Such TN concentrations, despite being relatively high, were also found in the sites with minimal human disturbance or set as nutrient criteria in several US ecoregions^48, 49^. In our study, in-stream TN concentration was determined by annual air temperature, % CaCO_3_, and % gravel. Temperature determines the speed of ecological processes, including N-fixation and litter decomposition in streams, resulting in a positive effect on TN concentrations^15, 50^. Soil lime (% CaCO_3_) content, originating from bedrock, may affect TN concentrations by determining alkalinity in waters that is negatively correlated to nitrification. Gravel content, representing rock fragments content of soils with particles larger than 2 mm, influences other soil properties including bulk density, porosity, and water infiltration and storage^51^. A soil with high % gravel has high drainage water, low understory cover and plant yield thereon^52^, and high erodibility^53^, resulting in a higher possibility of contributing nutrients to streams than other fine soil contents. Considering pH, annual precipitation had the most significant effects on it. The fact that the TGR region was suffered from acid precipitation explained the strongly negative relationship between annual precipitation and pH^54^. Annual temperature also had a negative influence on pH because nitrification speed may accelerate with temperature increase^50^. A positive relationship between % farmland and pH implies that salinization of soils caused by agricultural fertilization had influenced waters in this region. Farmland percent and most of the natural environmental variables also displayed direct impacts on diatom species composition. This result implies that both human and natural factors also had effects on diatoms through influencing other in-stream variables that were not monitored in the present study.

Diatom assemblages displayed distinct threshold responses to farmland and ISA in the watershed. A relatively high tolerance to agricultural disturbance was observed that substantial compositional changes occurred when farmland was higher than 25%. This threshold was in the range of agricultural thresholds identified by others. For instance, Waite found that the % low inorganic N diatoms in Eastern US streams showed a rapid decline at approximately 40% in watershed agricultural land use^26^. Allan proposed an agriculture threshold of 30–50% for streams to remain in good condition^19^. Feld found location-specific thresholds for lotic assemblages that several metrics changed markedly between 10–20% and 40–45% of arable lands for mountain and lowland streams, respectively^55^. Magierowski *et al*. detected a significant decline in Australian river health when grazing land use reached 37%^56^. The variation in agricultural thresholds among different studies originated from several aspects including concerned biological metrics, threshold methods, and whether or not to account for effects of natural factors on biological assemblages. Additionally, biotic responses to agricultural disturbance might be watershed-specific. Agricultural land use acts as a main source, while woodlands and wetlands are important sinks of non-point source pollution in the watershed. Therefore, influences of agricultural disturbance on biotic assemblages would be alleviated by woodlands and wetlands with effects determined by specific proportions and configurations in the watershed^57^. As for ISA thresholds, “no tolerance” responses were detected for different lotic assemblages. For instance, Smucker *et al*. detected ISA thresholds of 0.6–2.9% for lotic diatom metrics^25^. King *et al*. suggested a community-level ISA threshold of 0.68–1.28% for stream macroinvertebrates^58^. Hilderbrand and Utz suggested that most lotic sensitive taxa will be lost at approximately 3% ISA^59^. These low thresholds indicate that urbanization related land use has more significant impacts on lotic assemblages compared with agricultural land use, regardless of study locations or biological metrics. By comparison, agricultural disturbance may interact with natural factors in impacting lotic assemblages due to the fact that arable land is determined by regional natural conditions such as climate, soil, and geomorphology. We also found threshold responses of diatom assemblages to several natural environmental variables. This finding indicates that influences of natural factors on species compositions also should be considered for detecting accurate thresholds of human disturbance. Among the 13 diatom species that GF achieved the highest fitness, only *B. lanceolata* and *A. minutissimum* had low optima for human land use, while the other taxa had medium to high optima. This is because tolerant taxa are more responsive than sensitive taxa along environmental gradients. Most of these species also displayed similar optima for nutrient conditions.

Our findings have important implications for threshold identification and management practices. First, we found that among the four in-stream stressors in tributaries of TGR, only TP was principally influenced by human land use. This finding implies that TP can be effectively controlled by managing human activities. In contrast, efforts on reducing in-stream TN concentrations may be less effective due to relatively high TN background concentrations. Second, land use thresholds provide important evidence for watershed management. Specifically, to prevent considerable changes in species composition of lotic benthic diatoms in the TGR region, the agricultural area should not exceed ¼ of individual watershed areas. In addition, ISA should be strictly controlled in this agricultural region. During sampling, we observed that plenty of towns and other buildings were situated near stream banks without enough riparian buffers. This may be the reason for the disproportionate influence of the % ISA on lotic diatoms. Future planning and management should address the importance of restoring riparian zones to alleviate the disturbance of ISA^55, 60^. Our study on human land use thresholds does not imply that nutrient thresholds are not important. Nutrient thresholds provide causal evidence for prevent degradation of biotic conditions. However, nutrient thresholds may be impractical when the target nutrient is mainly influenced by natural factors and the background concentration is higher than the threshold. By comparison, human land use thresholds provide direct evidence for controlling anthropogenic degradation of biological conditions. Therefore, nutrient and land use thresholds are not mutually exclusive and both are valuable for management practices.

In conclusion, our study demonstrated the importance of detecting main sources of in-stream stressors *prior to* threshold estimation. Additionally, accounting for confounding effects of natural factors was essential when quantifying human land use thresholds. We suggest a detailed exploration of pathways through which human disturbance affects lotic ecosystems for defining reasonable and attainable ecological thresholds.
